# *Ralstonia pickettii* bloodstream infections with potential genomic link to internationally distributed contaminated saline solution, Germany, October 2023

**DOI:** 10.2807/1560-7917.ES.2024.29.3.2400010

**Published:** 2024-01-18

**Authors:** Manuel Krone, Vera Rauschenberger, Vera Blaschke, Heike Claus, Oliver Kurzai, Stefanie Kampmeier

**Affiliations:** 1 University Hospital Würzburg, Infection Control and Antimicrobial Stewardship Unit, Würzburg, Germany; 2 University of Würzburg, Institute for Hygiene and Microbiology, Würzburg, Germany; 3 Leibniz Institute for Natural Product Research and Infection Biology – Hans-Knoell-Institute, Jena, Germany

**Keywords:** *Ralstonia pickettii*, contamination, infusion solution, sepsis, infection control

## Abstract

*Ralstonia pickettii* is a Gram-negative rod which may cause invasive infections when they contaminate liquid medical products. After *R. pickettii* was detected in blood cultures and a stem cell product from three patients in a tertiary care hospital in Germany, whole genome sequencing of these three isolates and two water isolates from the environment was performed. Core genome multilocus sequence typing analysis showed that the three patient isolates were closely related and there was a large distance to the environmental isolates. In a genomic comparison, the patients’ isolates were distantly related to an *R. pickettii* strain from a cluster in Australia suspected to be caused by contaminated saline produced in India, while all liquid medical products with a link to all patients were produced in Europe or the United States. Our data point towards an ongoing risk by an unknown common source that could be traced back to medical products contaminated with *R. pickettii* and potentially distributed worldwide. Investigating invasive *R. pickettii* infections, identifying and testing medical products administered to the patients and timely whole genome sequencing may help identify the exact source of this potentially global outbreak.

## Background

*Ralstonia pickettii* is a Gram-negative, non-fermentative, aerobic rod that was first described in 1973. The pathogen was previously classified as *Pseudomonas pickettii*, later as *Burkholderia pickettii* [1]. It was renamed to *Ralstonia pickettii* in 1995. These bacteria grow in diverse water sources and soil. Although they are able to produce biofilms, their virulence is thought to be low [2,3]. Bloodstream infections (BSI) caused by *R. pickettii* have been reported since the 1980s, particularly in immunosuppressed or critically ill patients [2,4,5]. 

Contaminated medical products such as saline solutions, drug solutions or water were commonly identified as sources of infection [1,6,7]. Contamination of sterile products can occur during manufacturing processes because *R. pickettii* is able to bypass sterile filters with a pore size of 0.2 µm used to maintain sterility in pharmaceutical filling processes [8]. As bacteraemia with *R. pickettii* is very rare, nosocomial outbreaks with bacteraemia are often a reason for further investigation into the source of infection. From 2017 to 2022, no more than three detections of *R. pickettii* from blood cultures were reported per year from the whole country to the German Antibiotic Resistance Surveillance (ARS) [9]. In 2023, an outbreak of *Ralstonia mannitolytica* BSI in Italy was caused by contaminated urokinase which was produced in India, and an outbreak of *R. pickettii* BSI in India was caused by contaminated fentanyl [10,11].

## Outbreak detection

Within a 14-day period in October 2023, *R. pickettii* was detected in samples from three patients (one autologous stem cell (SC) product and two blood culture samples from BSI) treated at a tertiary care hospital in Germany without an obvious local epidemiological link (the three patients were hospitalised in different buildings without contact to each other or shared procedures). As this rate exceeds by far the baseline detection of *R. pickettii* species in clinical samples of the hospital (maximum of one positive sample per year during 2014–2022), an outbreak investigation was initiated on 20 October 2023.

## Methods

### Epidemiological investigations

Epidemiological investigations in the laboratory information system regarding detections of *R. pickettii* were performed by three infection control physicians (MK, VB, SK), a scientist (VR) and an infection control nurse (FH). Environmental sampling was performed by a nurse (FH). A nurse (FH) and a physician (MK) screened the patients’ files for clinical data, potential connections and exposures to medical products. Tracing back the applied solutions and medical products was performed by two physicians (MK, SK) together with pharmacists from the hospital’s pharmacy. They were supported by two physicians (VB, OK), two scientists (VR, HC) and technicians from the microbiological laboratory for microbiological analysis and sequencing. Bioinformatics analysis was performed by a scientist (HC) together with a physician (SK).

### Species identification and susceptibility testing

Standard blood culture bottles (BacT/ALERT FA Plus/FN Plus, bioMérieux, Marcy-l’Étoile, France) were incubated at 36 °C for up to 7 days. Blood culture bottles containing stem cell products (BacT/ALERT SA/SN, bioMérieux) were incubated at 30 °C (aerobic) and 36 °C (anaerobic) for 14 days. Water samples (100 mL) and saline samples (container volume, maximum 500 mL) were membrane-filtered and incubated on Trypticase soy agar (Thermo Fisher Scientific, Waltham, United States (US)) at 33 °C for 5 days. Subsequent species identification was performed by matrix-assisted laser desorption/ionisation time of flight-mass spectrometry (MALDI-TOF) using VITEK MS (bioMérieux). 

For antimicrobial susceptibility testing (AST), minimal inhibitory concentrations (MIC) were determined via gradient agar diffusion testing using MIC test strips (Liofilchem, Roseto degli Abruzzi, Italy) and interpreted according to the recommendations for non-species-related pharmacokinetic/pharmacodynamic breakpoints of the European Committee of Antimicrobial Susceptibility Testing (EUCAST) version 13.1 because species-specific breakpoints for *Ralstonia* spp. were not available [12].

### Typing based on whole genome sequencing 

To analyse the genetic relatedness of isolated *R. pickettii* strains, we applied whole genome sequencing using the Illumina NextSeq 2000 (Illumina Inc., San Diego, US). The genome sequences were assembled, and a core genome multilocus sequence typing (cgMLST) scheme for *R. pickettii,* with strain 52 (NCBI RefSeq accession number: GCF_002849525.1) as reference sequence, was created with the SeqSphere^+^ software version 9.0.12 (Ridom, Muenster, Germany). We compared alleles of coding regions in a gene-by-gene approach based on up to 2,154 target genes and visualised the results in a minimum spanning tree. For a more precise evaluation, a single nucleotide variant analysis based on 276,346 nt was performed and illustrated in a minimum spanning tree using the same software. 

## Results

### Case descriptions and epidemiological investigations

**Figure 1:**
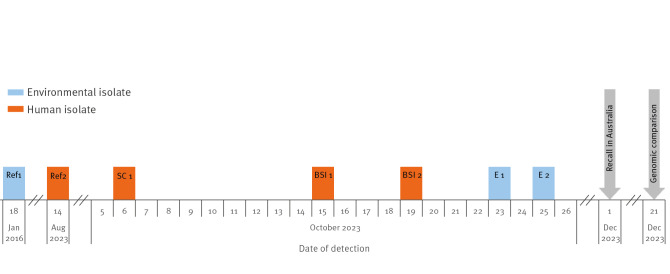
Timeline of the *Ralstonia pickettii* outbreak, Germany, October 2023 (n = 3)

BSI: blood stream infection isolate; E: environmental isolate; SC: stem cell material isolate; Ref: external reference strain.

In total, three patients were included in the here described *R. pickettii* outbreak. All of them (shown in a timeline in Figure 1) were immunocompromised and treated either in a stem cell transplantation or intensive care unit (ICU).

The Australian Therapeutic Goods Administration published a recall of saline products produced in India after contamination of this saline with *R. pickettii* was suspected on 1 December 2023 [13].

Patient 1 (SC1) was admitted to a peripheral ward for stem cell apheresis for an autologous stem cell transplantation. As the patient developed fever under neutropenia on day 2 of hospitalisation, two blood culture sets were drawn, and treatment with intravenous piperacillin/tazobactam was started until the day of the stem cell apheresis (day 7). Both blood culture sets remained sterile. *R*alstonia* pickettii* was detected in one of three stem cell products. The other two products remained sterile. The antibiotic treatment was switched to amoxicillin/clavulanic acid on the day of stem cell apheresis (day 7). In the 30 days before hospitalisation, the patient had had another three-day stay on the same ward.

Patient 2 (BSI 1) was transferred from a peripheral hospital to the anaesthesiological ICU. Three blood culture sets were sampled on day 2 of hospitalisation. *Ralstonia pickettii* was detected in all three aerobic blood culture bottles after 1 day of incubation. The anaerobic bottles remained sterile. The patient was treated with intravenous piperacillin/tazobactam but died on the day of sampling presumably due to underlying disease. Detailed information on the previous stay in the peripheral hospital was not available.

Patient 3 (BSI 2) was transferred to the neurological ICU from a peripheral hospital. After an increase in C-reactive protein up to 9.80 mg/dL (norm: 0–0.5 mg/dL) on day 3 of hospitalisation, three blood culture sets were sampled which remained negative. The patient developed fever on day 6. Therefore, three additional blood culture sets were sampled. In two of the three aerobic bottles (peripheral and from the central venous line), growth of *R. pickettii* was detected. In the aerobic bottle from the blood culture drawn from the arterial line, growth of *Staphylococcus epidermidis* was detected. The anaerobic bottles remained sterile. Clinically, the patient showed symptoms of ventilator-associated pneumonia. Antibiotic treatment with intravenous piperacillin/tazobactam was administered for 3 days, followed by 7 days of ceftriaxone. Under antibiotic treatment, the patient showed clinical improvement. On day 13, again three blood culture sets were sampled with the detection of *S. epidermidis* in the aerobic bottle drawn from the arterial line. All other bottles remained sterile. On day 26, the patient was transferred to a rehabilitation hospital.

The epidemiological investigation of the affected patients resulted in no link by place. In addition, neither healthcare workers nor any medical device were detected as common source of infection.

### Tracing back medical products

We further collected information from the three patients’ records in the electronic patient information system and the stem cell preparation protocols. The following solutions were identified as injected to all three patients or used for the preparation of intravenous solutions: 0.9% saline and an electrolyte solution. Furthermore, two of the patients had been injected with aqua ad iniectabilia, paracetamol and piperacillin/tazobactam including the patient without a previous stay in an external hospital. All products containing these solutions and used at the hospital were traced back in cooperation with the manufacturers. The solutions and the pharmaceutical products were produced in Germany, Italy, Spain, Portugal and the US. Information about the used products was communicated to the manufacturers, who started investigations but as yet, no contaminated product has been identified and investigations are still ongoing at the time of publication of this report.

The origin of solutions only applied to two patients (furosemide, norepinephrine, potassium chloride and propofol) or only applied to one patient (acid citrate dextrose, albumin, caspofungin, clonidine, dexamethasone, dihydralazine, dimenhydrinate, dimethyl sulfoxide, epinephrine, fosaprepitant, glucose, granisetron, hydrocortisone, ibuprofen, lormetazepam, metamizole, mesna, ondansetron, parenteral nutrition, remifentanil, urapidil, selenate, sodium bicarbonate, sufentanil, tris(hydroxymethyl)aminomethane, vasopressin, vitamin B1, vitamin B6, vitamin C) have so far not been included in the microbiological analysis and official report to the Federal Institute for Drugs and Medical Devices (BfArM), as a common source was assumed based on the genomic similarity of the patients’ isolates. 

We identified the invasive devices present during the time before the detection of *R. pickettii*: Patient 1 received a peripheral venous line at both in-patient treatments. Patient 2 was equipped with a central venous line, an arterial line and three peripheral venous lines. On admission, a fourth peripheral line was inserted. All catheters were present at death. No sampling was performed on the peripheral venous and arterial catheters and the central venous line of Patient 2. Patient 3 was, after transfer to our hospital, equipped with a new central venous line, an arterial line and a peripheral venous line. The peripheral line was removed on day 3 due to redness but no microbiological investigations were performed. The central venous line was removed on day 10 (4 days after the sampling of the positive blood cultures). The arterial line was removed on day 18.

### Microbiological sampling and analysis

To elucidate a potential source of infection within the water-supplying systems of the university hospital, we collected 85 environmental samples from 23 to 27 October 2023 from drinking and technical water, bronchoscopes, cell culture medium and surfaces, resulting in two *R. pickettii*-positive samples (E1 from water and E2 from cell culture medium) in late October. These were included in the subsequent genetic comparisons.

In the microbiological sample from the central venous catheter in Patient 2, we detected only few colonies of coagulase-negative staphylococci.

Nineteen saline products used on the wards where the patients were hospitalised (including the saline product used to wash the stem cells) were tested for microbial growth and remained sterile.

### Antimicrobial susceptibility testing

The AST showed the same susceptibility pattern in the isolates from Patient 1 and Patient 3 and a difference in cefotaxime susceptibility between those two and the isolate from Patient 2. The susceptibility profiles of the two environmental isolates differed for two of the tested antibiotics. All patient isolates differed from E1 in cefotaxime and meropenem susceptibility. Isolates from Patients 1 and 3 differed from E2 in cefotaxime and ciprofloxacin susceptibility, while the isolate from Patient 2 only differed from E2 in ciprofloxacin susceptibility (Table 1).

**Table 1:** Antimicrobial susceptibility testing results of *Ralstonia pickettii* isolates, Germany, October 2023 (n = 5)

	Patient 1 (SC 1)		Patient 2 (BSI 1)		Patient 3 (BSI 2)		E 1		E 2	
	MIC (mg/L)	Interpretation PK-PD	MIC (mg/L)	Interpretation PK-PD	MIC (mg/L)	Interpretation PK-PD	MIC (mg/L)	Interpretation PK-PD	MIC (mg/L)	Interpretation PK-PD
Ampicillin/sulbactam	0.023	S	0.032	S	0.016	S	1	S	0.38	S
Cefotaxime	0.75	S	1.5	I	0.75	S	3	R	1.5	I
Ceftazidime	16	R	24	R	16	R	32	R	24	R
Piperacillin/tazobactam	0.75	S	1.5	S	1	S	1.5	S	0.5	S
Meropenem	0.25	S	0,5	S	0.25	S	4	I	2	S
Gentamycin	256	R	> 256	R	256	R	0.75	R	128	R
Ciprofloxacin	0.19	S	0.094	S	0.19	S	0.094	S	128	R
Tigecycline	0.19	S	0.19	S	0.19	S	0.094	S	0.25	S

BSI: blood stream infection isolate; E: environmental isolate; MIC: minimal inhibitory concentration; SC: stem cell material isolate; PD: pharmacodynamic; PK: pharmacokinetic.

As there are no species-specific breakpoints for *Ralstonia* spp., interpretation is based on PK-PD breakpoints [12].

### Genomic comparisons

Due to an increase in the detection rate of *R. pickettii* in clinically relevant patient samples in Germany and a cluster of *R. pickettii* infections from Australia with a potential link to contaminated saline solutions [13], the national public health authority (Robert Koch Institute (RKI)) got in contact with us on 20 December 2023. The Australian Therapeutic Goods Administration had published a recall of saline products produced in India after contamination of this saline with *R. pickettii* was suspected on 1 December 2023 [13]. The recall was expanded to additional products from the same manufacturer on 18 December 2023 [14].

**Figure 2:**
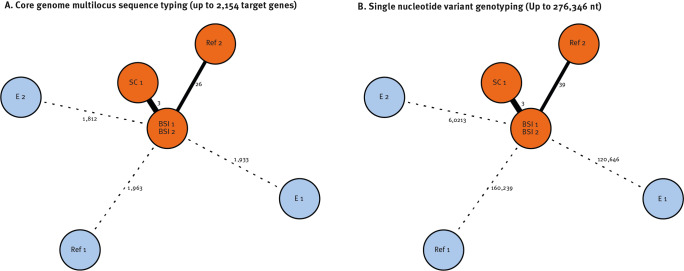
Minimum spanning trees comparing *Ralstonia pickettii* genome sequences derived from patient and water samples, Germany, October 2023 (n = 5)

BSI: blood stream infection isolate; E: environmental isolate; Ref: external reference; SC: stem cell material isolate.

On 21 December 2023, whole genome sequencing-based typing and genetic comparison of our patient isolates with internal environmental isolates and genome sequences submitted by other international laboratories resulted in one cluster and three singletons (Table 2 and Figure 2). The cluster comprised genotypes of strains derived from our in-house SC sample and the two BSI samples as well as the SAMN38255698 sequence submitted from Australia (Ref 2), which we assume to be at least distantly related to the German genotypes (26 alleles differences/39 nt) (Figure 2).

**Table 2:** Origin of *Ralstonia pickettii* isolates used for the genomic comparison analysis, Germany, October 2023 (n = 7)

Sample ID	Country	Collection date	Isolate name	Origination laboratory	Submitting laboratory	Authors
HY4146	Germany	15 Oct 2023	BSI 1	Institute for Hygiene and Microbiology, University of Würzburg		This report
HY4149	Germany	19 Oct 2023	BSI 2	Institute for Hygiene and Microbiology, University of Würzburg		This report
HY4115	Germany	6 Oct 2023	SC 1	Institute for Hygiene and Microbiology, University of Würzburg		This report
HY4163	Germany	23 Oct 2023	E 1	Institute for Hygiene and Microbiology, University of Würzburg		This report
HY4145	Germany	25 Oct 2023	E 2	Institute for Hygiene and Microbiology, University of Würzburg		This report
SAMN08239918	China	18 Jan 2016	Ref 1 (Assembly name: ASM284952; RefSeq Assembly ID: GCF_002849525.1)	State Key Laboratory for Diagnosis and Treatment of Infectious Diseases, The First Affiliated Hospital, School of Medicine, Zhejiang University		Zheng, B
SAMN38255698	Australia	14 Aug 2023	Ref 2 (= WGSID 23–013–0131)	New South Wales Health Pathology	Institute of Clinical Pathology and Medical Research, Westmead Hospital	Sullivan, G

BSI: blood stream infection isolate; E: environmental isolate; SC: stem cell material isolate.

Every circle represents one genotype, and numbers next to lines illustrate allele or nucleotide differences. For distance calculations pairwise missing values were ignored. Orange: human isolates; blue: environmental isolates.

## Outbreak control measures

The water supply system in which *R. pickettii* was identified during environmental sampling in the hospital was disinfected and not reused before additional samples returned negative for *R. pickettii*.

Results of the genotypic analyses were immediately communicated to the local, regional and national health authorities (RKI) and the BfArM on 22 December 2023. Following our report, the RKI requested, on 11 January 2024, microbiological laboratories in Germany to report all detections of *R. pickettii* to local health authorities and to send all *R. pickettii* isolates for genomic typing until 31 March 2024 [9]. Until the day of publication, no further *R. pickettii* were detected in our laboratory or – to the best of our knowledge – in other laboratories. In addition, the information was published that eight detections of *R. pickettii* from blood cultures from Germany were reported to ARS between September and November 2023 compared with zero to three reports per year in the years 2017 to 2022 [9].

## Discussion

Here, we report a cluster of three genetically related *R. pickettii* isolates originating from clinically relevant patient samples. This is particularly remarkable as no obvious epidemiological connection could be drawn, until genetic comparison with the strain from an Australian cluster indicated a potential relation. This suggests a common source outside the hospital which has not been fully clarified as yet. The investigations completed by 16 January 2023 point to saline or electrolyte solutions as the most probable candidates. This is consistent with the fact that *R. pickettii* infections are primarily caused by contaminated medical products [15-18], especially parenteral products, and it matches the results from the Australian cluster with a potential link to presumably contaminated saline solution from an Indian manufacturing plant [13]. As none of the intravenous products implicated in our epidemiological investigations was produced in this plant, an indirect connection of the presumed contaminations may be possible. Unfortunately, no genomes of isolates from previous *R. pickettii* outbreaks were publicly available, therefore we could not perform a genomic comparison with these clusters to identify potential links.

A pseudo-outbreak caused by contaminated growth media is very unlikely: Firstly, *R. pickettii* was identified in two different types of blood culture bottles (FA Plus for the blood cultures, SA for the stem cell product) and secondly, *R. pickettii* was found in three of three blood cultures from one patient and two of three blood culture sets from another patient, but was not detected in more than 1,000 blood cultures from other patients processed during the same month.

The difference in AST interpretation between the isolate from Patient 1 and the isolates from Patients 2 and 3 is the same as between Patient 1 and the environmental sample E2. This shows that AST was not reliable in detecting or ruling out links between bacterial isolates in this outbreak, as changes may happen rapidly in vivo or due to test variations. 

The present outbreak description has limitations. Firstly, the source of infection has not been identified yet. At the time of publication of this report, none of the saline solutions used in our three patients has been identified to have any links to the Indian manufacturer who is suspected to be source of the Australian cluster via contaminated saline. In addition, the company stated that they did not distribute saline solutions to the European continent. Secondly, as a public cgMLST scheme is not available, a threshold which discriminates between related and non-related genotypes is lacking. Hence, we had to compare genotypes relying on our in-house ad hoc cgMLST scheme. Nevertheless, during interpretation of genetic relatedness using a cut-off value, we relied on data published for *Burkholderia* spp. [19,20] due to genomic similarity and additionally performed a single nucleotide variant analysis for verification. As saline is frequently used as a solvent for drugs and to flush catheters the exact products and batch numbers applied for our patients were not documented; therefore, the batches we analysed microbiologically may not have been the same ones. As no further *R. pickettii* have been detected in our microbiological laboratory since November, the source may have been present only temporarily. Without identification, further cases elsewhere or a recurrence in our hospital may occur.

## Conclusion

We report genetically related *R. pickettii* isolates in multiple countries presumably caused by contaminated medical products. The variety of saline products and the global distribution hinders the identification of a source of contamination. When *R. pickettii* is detected in blood cultures or similar relevant clinical materials such as stem cells or central venous catheters, two things should be done: firstly, an analysis of the potential sources of infection should be carried out and secondly, genome sequencing and comparison with publicly available genomes should be performed to identify and eliminate the primary sources of the outbreak. International collaboration at the level of health authorities and microbiological diagnostic laboratories is essential for this.

## References

[1] Rajachandran K Varghese GS Kumar JV Mathew KT . Outbreak of nosocomial infection from an unusual source. Indian J Crit Care Med. 2022;26(9):1042-4. 10.5005/jp-journals-10071-24308 36213706 PMC9492743

[2] Tejera D Limongi G Bertullo M Cancela M . Ralstonia pickettii bacteremia in hemodialysis patients: a report of two cases. Rev Bras Ter Intensiva. 2016;28(2):195-8. 27410414 10.5935/0103-507X.20160033 PMC4943058

[3] Ryan MP Pembroke JT Adley CC . Ralstonia pickettii: a persistent gram-negative nosocomial infectious organism. J Hosp Infect. 2006;62(3):278-84. 10.1016/j.jhin.2005.08.015 16337309

[4] Baker MA Rhee C Tucker R Vaidya V Holtzman M Seethala RR Ralstonia pickettii and Pseudomonas aeruginosa bloodstream infections associated with contaminated extracorporeal membrane oxygenation water heater devices. Clin Infect Dis. 2022;75(10):1838-40. 10.1093/cid/ciac379 35594555

[5] Ryan MP Adley CC . Ralstonia spp.: emerging global opportunistic pathogens. Eur J Clin Microbiol Infect Dis. 2014;33(3):291-304. 10.1007/s10096-013-1975-9 24057141

[6] Ross B Steinmann J Buer J Dusse F Jakob H Schneemann H [Outbreak with Ralstonia pickettii caused by contaminated magnesium vials]. Dtsch Med Wochenschr. 2014;139(7):323-6. German. 24496893 10.1055/s-0033-1360059

[7] Moreira BM Leobons MB Pellegrino FL Santos M Teixeira LM de Andrade Marques E Ralstonia pickettii and Burkholderia cepacia complex bloodstream infections related to infusion of contaminated water for injection. J Hosp Infect. 2005;60(1):51-5. 10.1016/j.jhin.2004.09.036 15823657

[8] Sundaram S Auriemma M Howard G Jr Brandwein H Leo F . Application of membrane filtration for removal of diminutive bioburden organisms in pharmaceutical products and processes. PDA J Pharm Sci Technol. 1999;53(4):186-201. 10754712

[9] Koch-Institut R. Häufung von Ralstonia pickettii. [Increased occurrence of Ralstonia pickettii]. Epid Bull. 2024;2:20. German. Available from: https://www.rki.de/DE/Content/Infekt/EpidBull/epid_bull_node.html

[10] Fabricci M Trinca A Talotti L Busetti M Fotakis EA Merakou C A urokinase-associated outbreak of Ralstonia mannitolilytica bloodstream infections in haemodialysis patients in north-eastern Italy, January to April 2023. Euro Surveill. 2023;28(28):2300328. 10.2807/1560-7917.ES.2023.28.28.2300328 37440346 PMC10347894

[11] Mehra K Khera D Didel S Tak V . Outbreak of Ralstonia pickettii blood stream infection in pediatric intensive care unit. Indian J Pediatr. 2023. 10.1007/s12098-023-04973-3 38095782

[12] The European Committee on Antimicrobial Susceptibility Testing. Breakpoint tables for interpretation of MICs and zone diameters. Version 13.1. Växjö: EUCAST; 2023. Available from: ; http://www.eucast.org

[13] Department of Health and Aged Care. Recall: Logiwash and Legency Remedies sodium chloride 0.9% ampoules. Canberra: Australian Government; 2023. Available from: https://www.tga.gov.au/safety/product-recalls/recall-logiwash-and-legency-remedies-sodium-chloride-09-ampoules

[14] Department of Health and Aged Care. Safety alert: potential contamination of some saline products with Ralstonia pickettii. Canberra: Australian Government; 2023. Available from: https://www.tga.gov.au/news/safety-alerts/safety-alert-potential-contamination-some-saline-products-ralstonia-pickettii

[15] Chen YY Huang WT Chen CP Sun SM Kuo FM Chan YJ An outbreak of Ralstonia pickettii bloodstream infection associated with an intrinsically contaminated normal saline solution. Infect Control Hosp Epidemiol. 2017;38(4):444-8. 10.1017/ice.2016.327 28115025

[16] Lai HW Shen YH Chien LJ Tseng SH Mu JJ Chan YJ Outbreak of Ralstonia pickettii bacteremia caused by contaminated saline solution in Taiwan. Am J Infect Control. 2016;44(10):1191-2. 10.1016/j.ajic.2016.03.074 27424301

[17] Centers for Disease Control and Prevention (CDC) . Nosocomial Ralstonia pickettii colonization associated with intrinsically contaminated saline solution--Los Angeles, California, 1998. MMWR Morb Mortal Wkly Rep. 1998;47(14):285-6. 9572669

[18] Bedir Demirdag T Ozkaya-Parlakay A Bayrakdar F Gulhan B Kanik Yuksek S Suzuk Yildiz S An outbreak of Ralstonia pickettii bloodstream infection among pediatric leukemia patients. J Microbiol Immunol Infect. 2022;55(1):80-5. 10.1016/j.jmii.2020.12.004 33461864

[19] Lichtenegger S Trinh TT Assig K Prior K Harmsen D Pesl J Development and validation of a Burkholderia pseudomallei core genome multilocus sequence typing scheme to facilitate molecular surveillance. J Clin Microbiol. 2021;59(8):e0009321. 10.1128/JCM.00093-21 33980649 PMC8373231

[20] Appelt S Rohleder AM Jacob D von Buttlar H Georgi E Mueller K Genetic diversity and spatial distribution of Burkholderia mallei by core genome-based multilocus sequence typing analysis. PLoS One. 2022;17(7):e0270499. 10.1371/journal.pone.0270499 35793321 PMC9258848

